# Treatment of COVID-19 in Patients With Sarcoidosis

**DOI:** 10.3389/fmed.2021.689539

**Published:** 2021-07-16

**Authors:** Shreya Kondle, Titus Hou, Michael Manansala, Christian Ascoli, Richard M. Novak, Nadera Sweiss

**Affiliations:** ^1^Department of Medicine, University of Texas Southwestern Medical School, Dallas, TX, United States; ^2^Department of Medicine, University of Illinois College of Medicine at Rockford, Rockford, IL, United States; ^3^Department of Medicine, University of Illinois College of Medicine at Chicago, Chicago, IL, United States

**Keywords:** COVID-19, sarcoidosis, SARS-CoV-2, management, treatment, immunocompromised

## Abstract

Recent case reports and studies on treating COVID-19 in patients with chronic sarcoidosis describe different treatment modalities ranging from glucocorticoids to biologic medications. This review article summarizes seven case series and reports totaling 46 patients. While one case report suggested that sarcoidosis medications such as glucocorticoids may lengthen the COVID-19 disease course, another study with a larger registry suggests they do not. More studies are needed to elucidate an improvement in outcomes. It is possible that addition of TNF-alpha inhibitors at COVID-19 diagnosis decreases hospitalization rate. Overall, it is difficult to make firm conclusions regarding treatment given the heterogeneity of treatment modalities in the current literature. Our summarized findings are outlined with the opinions of sarcoidosis, pulmonary, and infectious disease experts in a flow chart that provides clinicians with our proposed management algorithm for sarcoidosis patients who develop COVID-19. We emphasize a need for exchange of information regarding management of COVID-19 in the setting of sarcoidosis to further improve treatment in this vulnerable population of patients.

## Introduction

Sarcoidosis is a multi-system inflammatory disease of unknown etiology. Most commonly, it presents as a pulmonary disease with bilateral hilar adenopathy, pulmonary reticular opacities, and non-caseating granulomas. However, it may present with cutaneous, ophthalmologic, musculoskeletal, cardiovascular, or CNS lesions. Though the archetypal patient in the United States is a middle-aged black female patient with hilar adenopathy, this characterization poorly captures the variety of findings in practice. For example, men are diagnosed earlier than women and Scandinavians are another common ethnic population to develop the disease (1). This is a multifaceted disease, and it is important to ground diagnostic inquiry on a patient's presentation.

Sarcoidosis is a diagnosis of exclusion with corresponding clinical and radiographic findings (2). Most patients do not need treatment as they have self-limiting, non-progressive disease. For those who need therapy, the first line treatment is glucocorticoids. Methotrexate with folate acid to reduce toxicity, azathioprine, leflunomide, TNF-a inhibitors, and mycophenolate may be used as steroid-sparing alternatives (3). Refractory sarcoidosis is treated with infliximab. Routine patient monitoring consists of both an examination of extrapulmonary involvement as well as follow-up on symptoms; patients on prednisone who are asymptomatic are often evaluated in 4–8-week intervals while those who are asymptomatic are seen at 3–4 month intervals (4).

Over the past year, a COVID-19 has swept the globe and presents new challenges to the management of pulmonary disease. Importantly, comorbidities indicated in COVID-19 deaths, hypertension, diabetes, cardiovascular disease, and respiratory system disease, are also comorbidities in patients with sarcoidosis (5, 6). Recent data suggests that sarcoidosis patients with decreased pulmonary function are at higher risk of adverse outcomes from COVID-19 (7). Therefore, identifying effective methods for treating patients with pre-existing sarcoidosis who are afflicted by COVID-19 is of great importance. In this article, we review recent case studies and reports on treating COVID-19 in patients with chronic sarcoidosis and provide a summary of existing research for medication adjustment for patients with sarcoidosis diagnosed with COVID-19. Of note, some patients included had extrapulmonary presentation of sarcoidosis, however this has yet to be identified as a significant risk factor for increased morbidity or mortality.

## Methods

**Figure 1:**
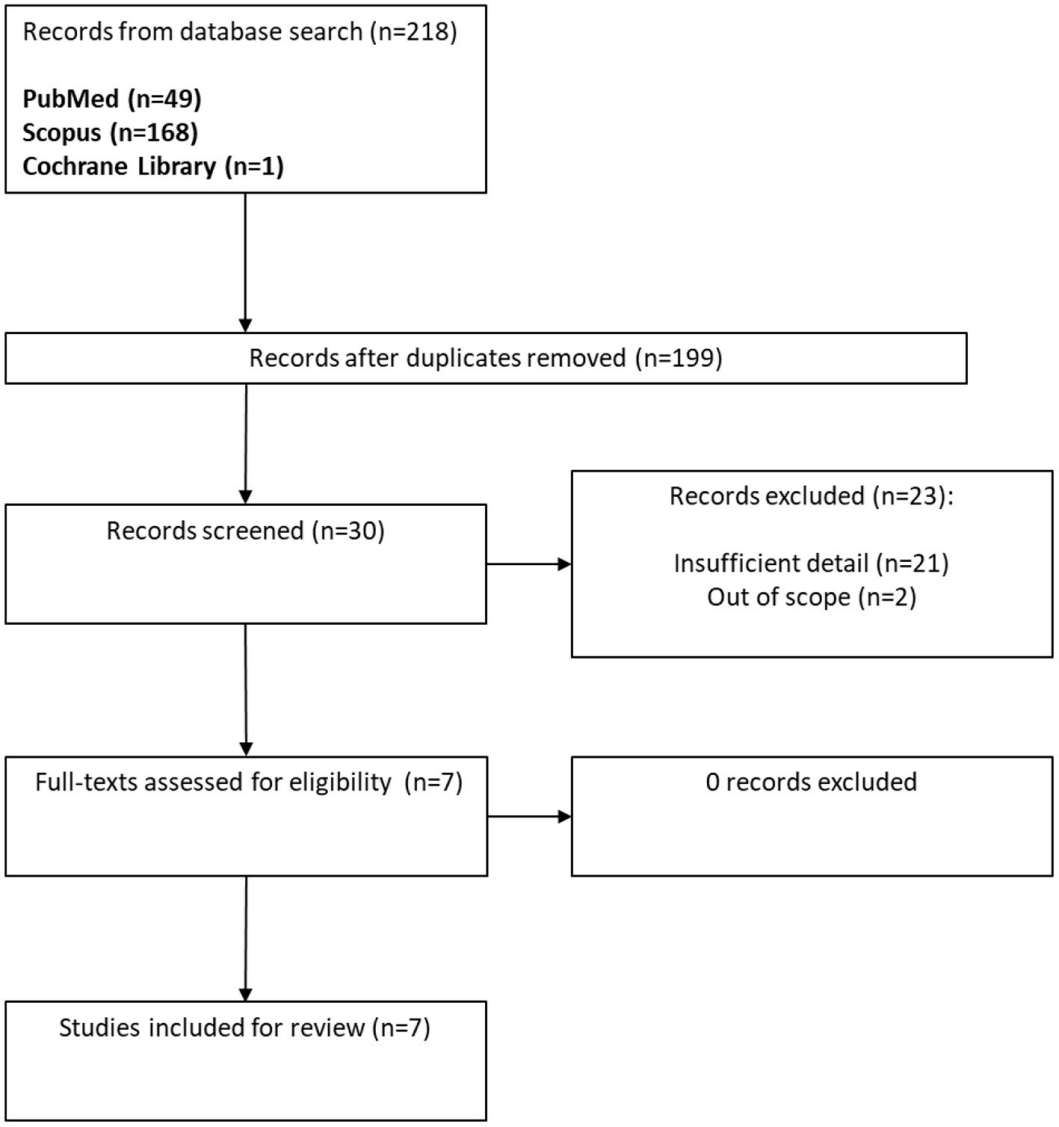
PRISMA flow diagram of literature search.

Until June 6th, 2021, the following search criteria with Boolean logic was utilized on the PubMed, Scopus, and Cochrane Library databases: (sarcoidosis) AND (Covid-19 OR SARS-CoV-2 OR Coronavirus disease 2019). Search results from the Scopus database were further limited to open-access articles in the subject area of Medicine and in the final publication stage. A total of 30 publications were identified and sorted by relevance. Inclusion criteria were case report or case series of patients with chronic sarcoidosis. Exclusion criteria were papers without mention of sarcoidosis baseline treatment, management of COVID-19, or COVID-19 outcomes (Figure 1). Seven publications totaling 46 patients were ultimately included in this review.

The seven publications reviewed had shared commonalities, which are reported in this paper, as well as differences which will be highlighted here. There is inconsistent data provided on existing sarcoidosis treatment and respective changes made to these medications. Many publications provide dosage and dosing schedule, while some only provide the type of medication. Case reports provided information such as the day of discharge relative to initiation of care while case series noted deaths, suspected cause of death, and other eventful outcomes such as bacterial infection. Further, there is a lack of systematic prospective studies and no control groups. There may be biases in the reporting of COVID-19 and its complications by patients and their primary physicians. Thus, data retrieved from tertiary centers may be biased. This heterogeneity in data reporting underscores the difficulty in drawing methodological conclusions from these publications and highlights the need for greater research on this subject matter. We recognize this as a limitation to our review and encourage further sharing of COVID-19 data in sarcoidosis patients.

## Results

Our literature review yielded case studies, case series, and guidelines for treating patients with COVID-19 superimposed on sarcoidosis. We have summarized the findings of seven case series and reports totaling 46 patients in Table 1. For sarcoidosis patients that do not require maintenance therapy, the current guidelines for COVID-19 appear to be acceptable (8, 9). However, for those taking disease modifying antisarcoid agents, there is a concern that their immunosuppressive regimen may have unwanted side effects of prolonging or worsening COVID-19 disease course. For example, the first line agent for sarcoidosis are glucocorticoids, but they have been linked to prolonged viral shedding–particularly in other respiratory viruses such as SARS (10). Sweiss et al. published guidance in April 2020 for the management of sarcoidosis amidst the COVID-19 pandemic in patients receiving immunosuppressive therapy (11). Currently, there is insufficient data to prove the immunologic benefits of continuing steroid and immunosuppressive medications for newly diagnosed COVID-19 positive patients suffering with chronic inflammation from rheumatological diseases other than sarcoidosis. Overall disease management was heterogenous in this review and it is difficult to draw firm conclusions from the current literature. However, this review does lend credence to this guideline (e.g., discontinuation of DMASD had good favorable outcomes) and highlights the need for further research on how best to manage patients with sarcoidosis who develop COVID-19.

**Table 1:** Summary of the literature review.

**Author, publication date**	**Case report or series** **[*N* = sarcoidosis patients]**	**Sarcoidosis history**	**Baseline sarcoidosis therapy**	**COVID-19 clinical presentation**	**Hospitalization status**	**Adjustment to sarcoidosis therapy**	**COVID-19 treatment**	**Outcome**
Bénézit, July 2020	Case Report	Pulmonary Sarcoidosis, well-controlled, diagnosed in 2015	HCQ (200 mg BID)	Fever (37.8')	Yes (day 16 of disease)	No change	Enoxaparin (60 mg) qd	Discharged (day 18 of disease)Day 40: mild asthenia, afebrile
Padala, July 2020	Case Report	Pulmonary and cardiac sarcoidosis, stable	MTX (20 mg) qwk ADM (40 mg) SC q2wk Prednisone (40 mg) qd, mexiletine, and amiodarone	Low-grade feversCoughMyalgia	Yes (day 7 of disease)	Discontinued: MTX and ADMPrednisone continued	HCQ (400 mg BID -> 200 mg BID) Tocilizumab 400 mg 1 dose Empiric ceftriaxone Vasopressors Mechanical ventilation (5 days) 2-4L O2 via nasal cannula	Discharged (day 16 of disease)
Györfi, October 2020	Case Report	Löfgren syndrome, well-controlled, diagnosed in 2019	No medications	Fever (38'C), dry cough, ankle pain at night and exerciseFever remitted on day 4 disease, but joint pain persisted	Yes (day 15 of disease)	Started: Prednisolone day 10 of disease for joint painDiscontinued: prednisolone day 17 of disease	HCQ (400 mg) qd PO	Uneventful disease course
Opoka, November 2020	Case Report	Pulmonary stage II sarcoidosis, well-controlled, diagnosed in 2018	No medications	N/A	Yes (day 7 of disease)	No changes	1.5 L/min O2 nasal cannula Enoxaparin (40 mg) qd SC Ceftriaxone (2 g) qd IV Levofloxacin (500 mg) BID PO Dexamethasone (6 mg) qd IV	Discharged (17 days of disease)
Yates, September 2020	Case series [1]	Pulmonary sarcoidosis diagnosed in 2011	Intermittent steroids	Intermittent coughDiarrhea	No	N/A	Doxycycline 100 mg BID for 10 days w/self-monitored pulse oximetry	Uneventful disease course
Jeny, October 2020	Case series [36]	Pulmonary [35]Intrathoracic lymph nodes [32]ILD [26]Lung Fibrosis [12]Skin [6]Peripheral lymph nodes [5]Liver [7]Heart [4]CNS [7]PNS [3]Kidney [3]Löfgren [1]	Long-term GC [25] MTX [8] Anti-TNF-α [6] HCQ [3] AZA [3] MMF [3]	Fever [24]Cough [29]SOB [24]Anosmia [8]Dysgeusia [7]NVD [10]RT-PCR+ [31]	Admitted [28] ICU [13]	1/25 discontinued GC4/8 discontinued methotrexate6/6 discontinued Anti-TNF-α	5/25 increased GC dose Additional GC [2] Antivirals [4] HCQ [5] Mechanical ventilation [4]	Death [5]Thrombosis [3]AKI [3]Bacterial infection [5]Discharges [31]
Manansala, November 2020	Case series [5]	African American [5]Pulmonary [2]Ocular cardiac [1]Neurologic [1]Testicular [1]	No treatment [2] Methylprednisolone (8 mg) qd [1] MTX (10 mg) qwk, HCQ (200 mg) qd, methylprednisolone (4 mg) qd [1] INX q8wks, MTX (7.5 mg weekly) [1]	Cough [4]Diarrhea [2]Fever [2]Myalgia [2]Dyspnea on exertion [1]SOB [1]Anosmia [1]Dysgeusia [1]	ICU [2]	No change	2/5 no treatment 3/5 HCQ and azithromycin 1/5 tocilizumab 1/5 prednisone	Death [1], likely from thromboembolic eventDischarged [1]

*ADM, adalimumab; GC, glucocorticoid; HCQ, hydroxychloroquine; INX, infliximab; MTX, methotrexate; MMF, mycophenolate mofetil; N/A, not applicable*.

## Discussion

### Risk Factors of Worsening Outcomes

An examination of 7,337 COVID-19 patients from a hospital system in New York City found 37 people with concomitant sarcoidosis. Of this population, 14 had moderate to severely impaired pulmonary function with nine succumbing to COVID-19 whereas only three of the 23 without these physiologic deficits succumbed to COVID-19. This data shows those diagnosed with COVID-19 with decreased pulmonary reserve increases the risk of adverse outcomes, measured by increased rates of intubation and hospital mortality (*p* = 0.003) (7). The articles reviewed do not show a worse or better management strategy since all case reviews had no mortalities and it was unclear which COVID-19 treatment was offered in 5 of 6 deaths occurring in the case series. Comorbidities in the deaths included uncontrolled hypertension, uncontrolled diabetes, active smoker, hemodialysis, and chronic kidney disease.

### Use of Glucocorticoids

A series of correspondences published in the Annals of Rheumatic Diseases discussed the role of corticosteroid modification in the treatment of patients with sarcoidosis who developed COVID-19 and later highlighted potential protective qualities of TNF-alpha antagonists. The first correspondence by Györfi et al. reported a case of COVID-19 relapse in a patient with Löfgren syndrome diagnosed in 2019 after apparent resolution of COVID-19 and a negative COVID-19 PCR test 14-days after exposure (12). 5 days after initiation of prednisolone treatment for joint pain, the patient developed pneumonia, suggesting that the glucocorticoids may have caused immunosuppression enabling SARS-CoV-2 to re-attack the patient's body. In one response, Jeny et al. reviewed 36 patients with sarcoidosis across 15 French hospital centers who developed COVID-19. Twenty five patients were taking corticosteroids at the time of diagnosis (13). One patient with long-term corticosteroid treatment stopped their treatment following COVID-19 diagnosis, five patients had an increased dosage in their treatment, and two patients without previous treatment with steroids were started corticosteroids. Eleven of the patients initially taking corticosteroids at the time of COVID-19 diagnosis were admitted into the ICU. It is unclear whether the five deaths in this data had a history of corticosteroid use for sarcoidosis, however, the authors conclude that glucocorticoid use do not worsen COVID-19 disease course.

Among non-sarcoidosis patients, there is evidence that long-term glucocorticoid treatment ≥10 mg/d may play a role in worsening COVID-19 outcomes among patients with systemic lupus erythematosus (14). In a study of COVID-19 patients with chronic immune-mediated inflammatory arthritis by Favalli et al. treatment with glucocorticoids displayed an increased risk of COVID-19 infection (15). After broadly examining a cohort with rheumatic disease, Strangfeld et al. urges caution in increasing glucocorticoid dosage, suggesting higher dosage increases the likelihood of COVID-19 death (16). For this reason, it is immensely important to further investigate the role of introducing or continuing prior steroid usage following COVID-19 diagnosis.

### Use of TNF-Alpha Antagonists

Jeny et al. also discussed possible protective qualities that prior TNF-alpha treatment may have had on sarcoidosis patients with a new COVID-19 diagnosis. Six patients with prior TNF-alpha antagonist treatment had their treatment suspended to mitigate further immunosuppression. Of the six, one patient died of hypercapnia secondary to chronic obstructive pulmonary disease and obesity and the rest were not admitted to the ICU during their disease course. Consistent with previously published data, the severest forms of COVID-19 in Jeny et al.'s data were not associated with prior TNF-alpha antagonist treatment (17–19). In a case series of 5 patients, Manansala et al. had one patient with testicular sarcoidosis on anti-TNF therapy who had a limited COVID-19 course without COVID-19 treatment (20). Among inflammatory bowel syndrome patients who are infected with COVID-19, these inhibitors have comparable or better outcomes than other non-biologic disease-modifying antirheumatic drugs (DMARDs) (21).

### Use of Biologics With Glucocorticoids

Padala et al. presents a unique case of a pulmonary and cardiac sarcoidosis patient with a varied immunosuppressive home regimen prior to COVID-19 diagnosis: 40 mg biweekly subcutaneous adalimumab, 40 mg daily prednisone, and 20 mg weekly methotrexate (22). In April 2020, guidelines by the American College of Rheumatology (ACR) stated to avoid abruptly stopping glucocorticoid treatment for COVID-19 patients with rheumatologic disease regardless of infection status and to discontinue JAK inhibitors, non-IL-6 biologics, leflunomide, immunosuppressants, and methotrexate (23). Therefore, Padala et al. continued the patient's prednisone and held the patient's methotrexate and adalimumab (22). The patient's clinical state deteriorated leading to the use of vasopressors, mechanical ventilation, administration of hydroxychloroquine, empiric ceftriaxone, and a single dose of tocilizumab. However, the patient eventually recovered. This report suggests that inclusion of prednisone in the treatment protocol may have unique anti-inflammatory effects that work in combination with tocilizumab despite delays in viral shedding time glucocorticoids. Manansala et al. also reported on a patient wo received tocilizumab and hydrocortisone, however, patient died from pulmonary emboli (20). The varied results highlight the need for greater study of this treatment modality.

### Use of Antimalarials

There was debate on the usage of antimalarials in the treatment of COVID-19. Yates et al. described a case series which included the administration of doxycycline to four COVID-19 patients with varying medical history (24). A patient who was diagnosed with pulmonary sarcoidosis in 2011 treated with intermittent steroids was prescribed 100 mg doxycycline twice daily with pulse oximetry monitoring following COVID-19 diagnosis. A transient decrease in oxygen saturation led to a visit to the emergency department, where the patient's oxygen saturation level normalized and the patient was discharged without additional treatment and he returned home to complete a 10-day course of doxycycline. Bénézit et al. presented a case report of a French pulmonary sarcoidosis patient diagnosed in 2015 with long-term hydroxychloroquine treatment prior to COVID-19 diagnosis, which was continued throughout his disease course (25). The patient required hospitalization after symptoms progressed to constant shortness of breath and thoracic pain. The patient was administered enoxaparin at discharge to mitigate thromboembolic events. Though this patient recovered from COVID-19 while taking hydroxychloroquine, numerous studies, including a randomized control trial, have since demonstrated that hydroxychloroquine does not provide protection against COVID-19 nor limit disease course (18, 26–28). Clinical trials are ongoing to elucidate the effectiveness of doxycycline although the British clinical trial PRINCIPLE has found no reduction in recovery time or hospitalization rate in their doxycycline arm compared to the standard COVID-19 treatment arm (29–31).

## Conclusion

**Figure 2:**
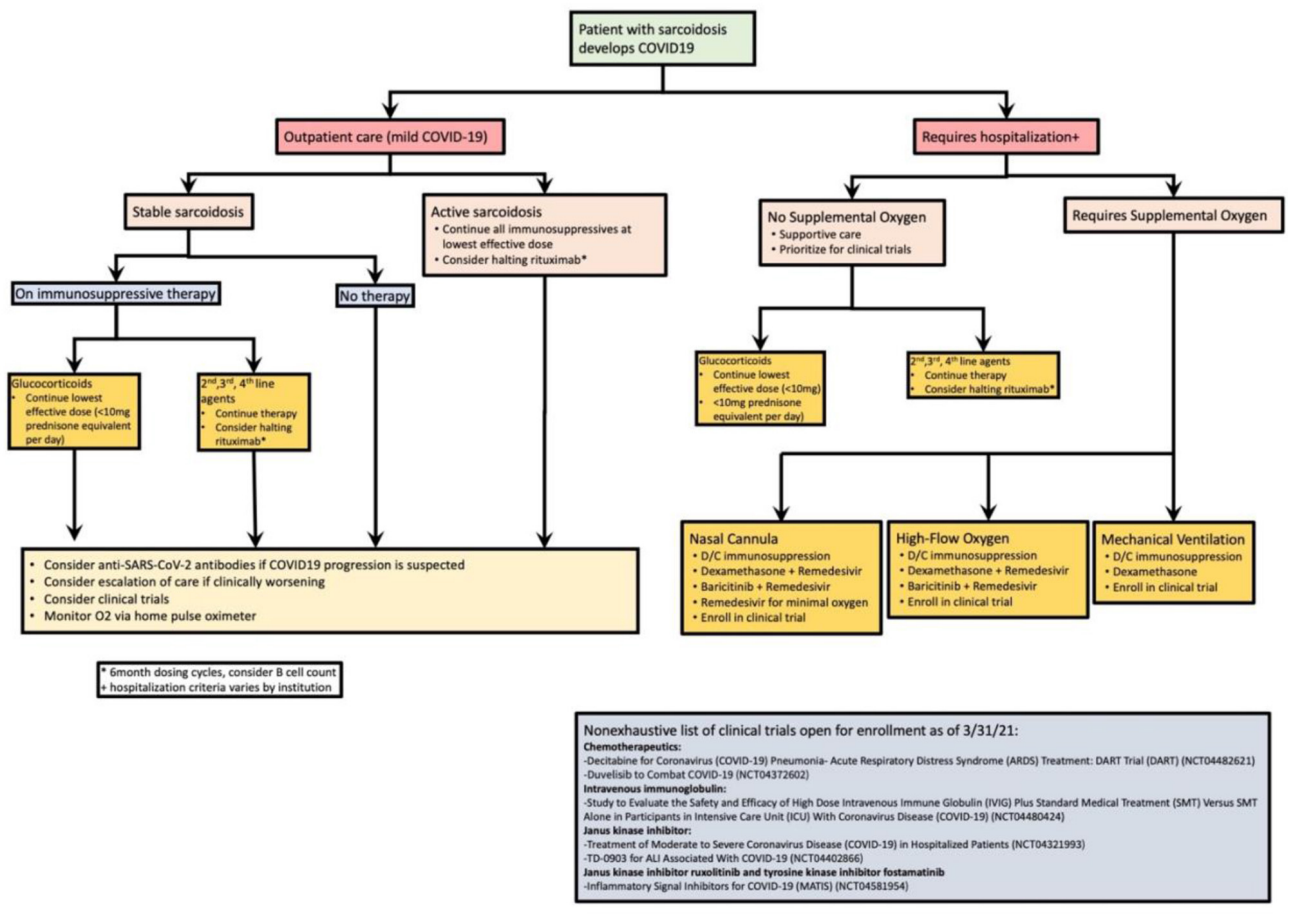
Proposed management algorithm of sarcoidosis patients who develop COVID-19. Active sarcoidosis is based off index organ requiring treatment. Our approach divides outpatient and inpatient care. This figure is a summation of expert advice, NIH recommendations, and the case series/reviews highlighted in this study.

For over a year, COVID-19 has presented a novel challenge for patients with chronic lung disease due to the respiratory disease pathology caused by SARS-CoV-2. We recognize that in the pandemic, the science for managing patients with sarcoidosis is rapidly evolving and that new findings may offer clearer management guidance. Here, we offer a suggestion in management (Figure 2) based on NIH guidelines and our findings from this review (9). The medication that some people take, such as glucocorticoids, may lengthen the disease process (10). This article summarizes seven case series and reports totaling 46 patients who have had a chronic diagnosis of sarcoidosis and a subsequent COVID-19 infection. Some patients had chronic medications to manage their symptoms while others did not. These studies suggest that use of TNF-alpha inhibitors at the time of COVID-19 diagnosis may result in a decreased rate of hospitalization. Additionally, in one case, glucocorticoids appeared to have worsened a patient's disease process (12). However, registry data suggests that glucocorticoids for symptomatic control of sarcoidosis may not improve or worsen outcomes for subsequent SARS-CoV-2 infection, but larger sample sized studies are needed (32, 33). A major limitation of this study is the lack of large randomized control trials and high-quality evidence for treating COVID-19 in sarcoidosis, highlighting the need for further research in this arena. Given the use of dexamethasone in the critically ill, empirical use of corticosteroids may continue to be the best guidance available. Additionally, this article underscores the importance of global information exchange for rare disease, such as sarcoidosis, so that clinicians can better provide evidence-based medical management and improve their patient's outcomes.

## Author Contributions

SK and TH wrote the bulk of the manuscript. MM worked with SK and TH to edit the contents of the paper as well as contributed several paragraphs of the manuscript. CA provided pulmonary disease expertise as well edited the contents of the manuscript. RN provided infectious disease expertise as well as edited the contents of the manuscript. NS provided expertise in sarcoidosis as well as edited the contents of the manuscript. All authors contributed to the article and approved the submitted version.

## Conflict of Interest

The authors declare that the research was conducted in the absence of any commercial or financial relationships that could be construed as a potential conflict of interest.
